# Development and evaluation of prompts for a large language model to screen titles and abstracts in a living systematic review

**DOI:** 10.1136/bmjment-2025-301762

**Published:** 2025-07-22

**Authors:** Ava Homiar, James Thomas, Edoardo G Ostinelli, Jaycee Kennett, Claire Friedrich, Pim Cuijpers, Mathias Harrer, Stefan Leucht, Clara Miguel, Alessandro Rodolico, Yuki Kataoka, Tomohiro Takayama, Keisuke Yoshimura, Ryuhei So, Yasushi Tsujimoto, Yosuke Yamagishi, Shiro Takagi, Masatsugu Sakata, Đorđe Bašić, Eirini Karyotaki, Jennifer Potts, Georgia Salanti, Toshi A Furukawa, Andrea Cipriani

**Affiliations:** 1 Department of Psychiatry, University of Oxford, Oxford, UK; 2 Division of Clinical Informatics, Beth Israel Deaconess Medical Center, Boston, Massachusetts, USA; 3 University College London, London, UK; 4 Vrije Universiteit Amsterdam, Amsterdam, The Netherlands; 5 Psychology & Digital Mental Health Care, Technical University of Munich, Munchen, Germany; 6 Psychiatry and Psychotherapy, Technical University of Munich School of Medicine, Munich, Germany; 7 Department of Clinical, Neuro and Developmental Psychology, Vrije Universiteit Amsterdam, Amsterdam, The Netherlands; 8 Technical University of Munich, Munich, Germany; 9 Kyoto Min-iren Asukai Hospital, Kyoto, Japan; 10 Kyoto University, Kyoto, Japan; 11 Okayama Psychiatric Medical Center, Okayama, Japan; 12 The University of Tokyo, Bunkyo, Tokyo, Japan; 13 Independent Researcher, Kyoto, Japan; 14 Nagoya City University Graduate School of Medical Sciences and Medical School, Nagoya, Japan; 15 NIHR Oxford Health Biomedical Research Centre, Oxford, UK; 16 University of Bern, Bern, Switzerland; 17 Kyoto University Graduate School of Medicine Faculty of Medicine, Kyoto, Japan

**Keywords:** Data Interpretation, Statistical, Machine Learning, PSYCHIATRY

## Abstract

**Background:**

Living systematic reviews (LSRs) maintain an updated summary of evidence by incorporating newly published research. While they improve review currency, repeated screening and selection of new references make them labourious and difficult to maintain. Large language models (LLMs) show promise in assisting with screening and data extraction, but more work is needed to achieve the high accuracy required for evidence that informs clinical and policy decisions.

**Objective:**

The study evaluated the effectiveness of an LLM (GPT-4o) in title and abstract screening compared with human reviewers.

**Methods:**

Human decisions from an LSR on prodopaminergic interventions for anhedonia served as the reference standard. The baseline search results were divided into a development and a test set. Prompts guiding the LLM’s eligibility assessments were refined using the development set and evaluated on the test set and two subsequent LSR updates. Consistency of the LLM outputs was also assessed.

**Results:**

Prompt development required 1045 records. When applied to the remaining baseline 11 939 records and two updates, the refined prompts achieved 100% sensitivity for studies ultimately included in the review after full-text screening, though sensitivity for records included by humans at the title and abstract stage varied (58–100%) across updates. Simulated workload reductions of 65–85% were observed. Prompt decisions showed high consistency, with minimal false exclusions, satisfying established screening performance benchmarks for systematic reviews.

**Conclusions:**

Refined GPT-4o prompts demonstrated high sensitivity and moderate specificity while reducing human workload. This approach shows potential for integrating LLMs into systematic review workflows to enhance efficiency.

## Introduction

 Systematic reviews play an essential role in synthesising research findings and developing guidelines for clinical and research practices.^1 2^ However, while biomedical research is rapidly evolving, the output of systematic reviews can be labourious and become outdated quickly.^1 3^ Living systematic reviews (LSRs) have become a popular way of providing a constantly updated synthesis of new evidence.^1 4^ While LSRs ensure timeliness, the repeated screening of new references adds a significant workload for human reviewers.^5^

Large language models (LLMs) such as GPT-4 can automate certain tasks in generating and processing texts.^6^ These models are trained on text data which, allow them to understand context and semantic relationships, as well as extract relevant information from various datasets.^7^ Recent studies using LLMs for screening records reported promising preliminary results, with sensitivity ranging from 95% to 100% and specificity between 70% and 90%, suggesting their potential to complement human efforts.^8 9^ However, these studies also identified limitations, such as inconsistent handling of nuanced inclusion criteria, difficulty in distinguishing borderline cases and susceptibility to bias from training data. For instance, models may prioritise randomised controlled trials over observational studies if the former were more common in their training data, even when both are eligible.^10^ Even the highest reported performance scores may not be sufficiently accurate for reviews requiring near-perfect recall, such as those in medical interventions or regulatory contexts, where missing relevant studies could have critical consequences.^10 11^

As part of the project to conduct a series of LSRs (Global Alliance for Living Evidence on aNxiety, depressiOn and pSychosis), this study aimed to evaluate the efficacy of using an LLM in the Evidence for Policy and Practice Information (EPPI) Reviewer^12^ platform to screen papers for inclusion at the title/abstract phase of an LSR on prodopaminergic interventions for anhedonia.^13^

### Research objectives

1. Design a high-accuracy workflow that reduces the workload for human reviewers by using the most recent LLMs for screening titles and abstracts in systematic reviews.
2. Develop optimal prompts through prompt engineering (the process of designing effective instructions for an LLM to perform a specific task) to screen a set of papers for the LSR.
3. Evaluate the performance of the LLM to inform a decision as to whether the LLM should be used in subsequent updates of the living review.

### Research questions

1. How can a method be developed to create high-performing prompts that enable unbiased evaluation of their performance?
2. What is an effective approach to developing prompts for the LLM to correctly include/exclude a paper in a specific LSR?
3. What is the internal and external validity of the LLM models in comparison with the judgements completed by human screeners?
4. Does the LLM provide consistent responses when applied multiple times to the same records?

## Methods

### Study design

This study reports the refinement, testing and validation of prompts to use an LLM for screening titles and abstracts in an LSR of prodopaminergic interventions for anhedonia.^13^ Prescreened data from the baseline LSR and two updates comprised the dataset we used for evaluation (see next section). As described below, the baseline LSR was split into ‘development’ and ‘test’ sets, and data from the two review updates were used to validate the prompts (the ‘validation set’). Prompts were developed on the ‘development’ set and, once performance was satisfactory, their performance was tested on the ‘test’ set. Finally, the same prompts were evaluated against the validation set.

### Dataset

The dataset consisted of records from the baseline living review and two updates, as summarised in table 1. The table shows the number of records that were assessed by human reviewers at two stages of screening. First, records were assessed based on their titles and abstracts. Those records that could not be excluded based on the information provided in their titles and abstracts were classified as ‘included at title/abstract’ and their full texts were retrieved. These full-text reports were assessed and the records were either ‘included at full text’ or excluded. The number of records without abstracts was significant, as these would need to be screened manually, might require obtaining their full texts for assessment, and therefore could not benefit from the use of the LLM screening workflow reported here. While they were not included in our evaluation of LLM screening, they are accounted for when estimates of workload reduction are calculated.

**Table 1:** Summary of gold standard data for testing and validation

	No abstract	Used for prompt development*	Included at title/abstract	Excluded at title/abstract	Included at full text	Excluded at full text	Total
Baseline	1151	1045	290	10 498	60	230	12 984
Update 1	7	0	1	159	1	0	167
Update 2	9	0	12	313	2	10	334

* All prompt development records were drawn from the baseline dataset.

### Development and refinement of prompts; and their final validation

In a typical supervised machine learning workflow, records are split into ‘train’ and ‘test’ sets, with the test set used to evaluate the performance of a machine learning model that has been built with the ‘train’ set. (Please note, as we are developing prompts in this study rather than training a new model, the equivalent of a ‘train’ set in our use case is termed a ‘development’ set.) In addition, a third ‘validation’ set is often used, to test the machine learning model on a new (often ‘real world’) dataset. This structure thus addressed threats to internal validity, using the test dataset, and external validity, using the validation dataset.

In the case of using an LLM for screening, no new machine-learning model is built. Instead, ‘prompts’ are developed and refined and then used in a large general language model. This process still requires the careful separation of records into two sets: those used for developing and refining the prompts, and those used for testing their accuracy. Once developed, the prompts can be validated in the same way as a conventional machine learning model, using a closely related dataset. Due to the novelty of the research and the lack of established methods for developing and testing prompts in LSRs, the first objective was to develop and pilot a workflow that can use LLMs to reduce manual screening efforts in this context.

At the outset of this work, we did not know how much data would be needed to develop and refine the prompts. For supervised machine learning, it is usual to take most of the records (80–90%) for training the model, leaving a much smaller subset for testing. A potential benefit of using the LLM is that a new model does not need to be built, and a much smaller proportion of the data is used for developing prompts. This potentially leaves a much larger proportion of available data to be used to test prompt performance.

**Figure 1:**
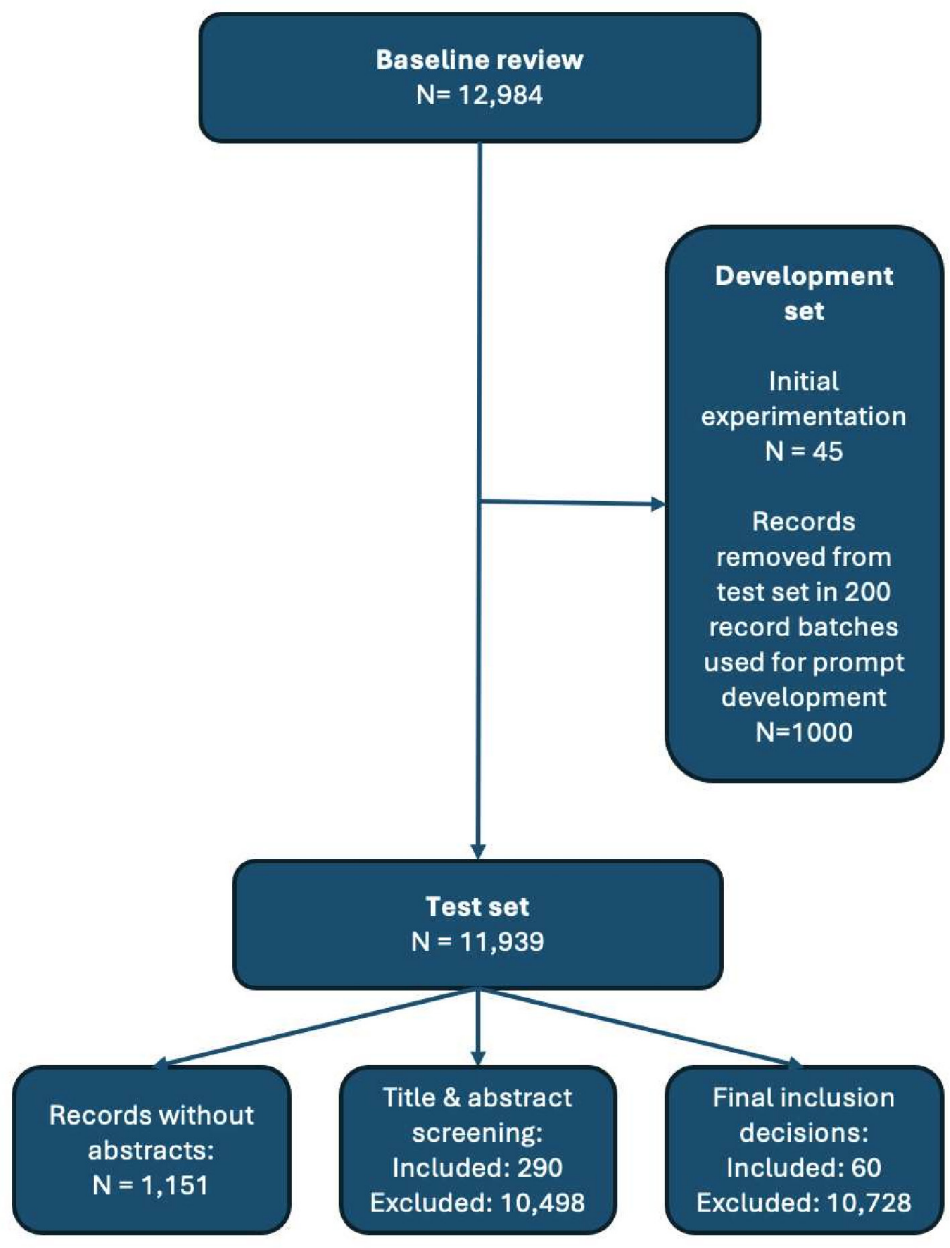
Creation of development and test sets of records in the baseline review.

We therefore developed a workflow to incrementally develop and test prompts before final testing (see figure 1). To begin with, all bibliographic records were considered part of the ‘test’ dataset, which was reserved to test prompt performance. Data to develop and refine prompts were removed at random from the test dataset and placed in the ‘development’ dataset—and not replaced. After an initial batch of 45 records for initial experimentation, these records were removed in batches of 200. In the first batch of 200, we oversampled from records that had been ‘included’ at the title and abstract stage by humans, as the prevalence of relevant records was low, and we needed to develop prompts that found them reliably. We therefore randomly selected 50 relevant records and 150 irrelevant ones in the first batch of 200. Thereafter, we sampled at random, yielding a ‘relevant’ prevalence rate of 17%.

We tested and refined our prompts on 50 records at a time within each batch to keep numbers manageable and because this number seemed to provide sufficient variation for incremental testing. We assessed accuracy as ‘sufficient’ only if 100% of relevant records were identified by the LLM. This process continued until either accuracy was sufficient or further iterations did not improve performance. At last, final prompts were tested using the remaining records in the test dataset, and overall performance statistics were calculated. After the prompts were developed, refined and tested on the baseline review, they were validated against two updates of the LSR.

The Population, Intervention, Comparator, Outcome (PICO) framework from the LSR was used to create prompts that aimed to determine the inclusion and exclusion of records (table 2). The software platform EPPI Reviewer was used to manage data, and prompt development and testing. It uses the OpenAI GPT-4o Application Programming Interface (API) via Azure (V.2024-02-01), enabling us to use the same system for this investigation as was used for manual title and abstract screening. Parameters in the API were set to minimise variation, so ‘temperature’ (‘randomness’) was set to zero, and ‘top p’ (the minimum probability threshold for an output) was set to 0.95. (For a detailed description of the prompt structure in EPPI Reviewer and the coding system, see online supplemental appendices A and B.)

**Table 2:** PICO questions and prompt development

PICO	PICO element	Base prompt	Modified prompt	Final prompt
Intervention	Include → pharmacological intervention involving dopamine	NOT pharmacological and dopamine → non_pharmacological_dopamine → this study does NOT report an evaluation of pharmacological interventions involved with dopamine pathways	non_pharmacological_dopamine: boolean // return TRUE if this abstract does NOT report an evaluation of a pharmacological intervention involved with dopamine pathways	non_pharmacological_dopamine: boolean // return TRUE if this abstract does NOT report an evaluation of a pharmacological intervention (including phelezine or imipramine) involved with dopamine pathways
Population	Include → human participants	NOT human study → not_human_study: boolean // this study does NOT report human participants	not_human_study: boolean // return TRUE if this abstract does NOT report a study conducted with human participants	not_human_study: boolean // return TRUE if this abstract does NOT report a study conducted with human participants
Population	Include → participants with unipolar depression	NOT depression → not_depression: boolean // this study does not report participants with depression	not_depression: boolean // return TRUE if this abstract does NOT report a study among participants with depression	not_depression: boolean // return TRUE if this abstract does NOT report a study among participants with depression (including chronic depression)
Study design	Include → randomised controlled trials	NOT RCT → not_rct: boolean // this study is not a randomized controlled trial	not_rct: boolean // return TRUE if this study is a randomized controlled trial	not_rct: boolean // return TRUE if this study is NOT a randomized controlled trial
Study design	Exclude → systematic reviews	Systematic review → systematic_review: boolean // this abstract reports the results of a systematic review	systematic_review: boolean // return TRUE if this abstract reports the results of a systematic review	systematic_review: boolean // return TRUE if this abstract reports the results of a systematic review
Population	Exclude → schizophrenia diagnosis	Diagnosis scz → diagnosis_of_schizophrenia: boolean // this study focuses on people with a diagnosis of schizophrenia	diag_scz: boolean // return TRUE if this study focuses on people with a diagnosis of schizophrenia or psychosis	diagnosis_of_schizophrenia: boolean // do the participants in this study have an explicit diagnosis of schizophrenia or psychosis?
Population	Exclude → postpartum depression	Diagnosis ppd → diagnosis_of_post_partum_depression: boolean // this study focuses on people with a diagnosis of postpartum depression	diag_ppd: boolean // return TRUE if this study focuses on women with postpartum depression	diagnosis_of_post_partum_depression: boolean // do the participants in this study have an explicit diagnosis of postpartum depression?
Control intervention	Include → placebo controlled	NOT placebo-controlled → no_placebo: boolean // this study does not include a placebo group	no_placebo: boolean // return TRUE if there is no mention of a placebo group and no mention of “yesPlacebo” in this abstract	no_placebo: boolean // return TRUE if this abstract does NOT report a placebo or comparison group (or mention of yesPlacebo)

### Performance metrics and statistical analysis

To assess the accuracy of the outputs created by the LLM compared with manual screeners, we calculated sensitivity, specificity, accuracy and workload reduction. Given that our primary concern is the impact of using artificial intelligence (AI) on review findings, sensitivity is calculated according to whether any records that were ultimately included in the review were lost. We also calculate sensitivity in terms of records that reviewers deemed necessary to check at full text. Workload reduction was calculated as the number of records that had been screened based on their titles and abstracts and that the LLM excluded. Thus, all records that did not have an abstract were assumed to require manual assessment and included as such in this calculation. Workload reduction does not account for the considerable time that it took to develop and refine the prompts.

For descriptions of performance metrics, see online supplemental appendix C.

### Consistency of output

As LLMs do not always generate identical outputs when presented with the same inputs, it is important to understand whether this can affect accuracy—and to what extent. To evaluate the consistency of the output, the team entered the final prompts for the same set of 50 randomly selected records 10 times. This was calculated using the number of times the LLM gave the correct answer (ie, agreed with the human screeners) divided by the number of times the data was run through the prompts. The team referred to previous works that have considered an accuracy score of 80–95% as ‘on-par’ with human reviewers, scores of 60–80% as ‘near-par’ and scores below 60% as ‘subpar’.^14 15^

## Results

### Iterative optimisation of prompts

The initial development and refinement of prompts used 1045 records, and the prompts were then tested on the remaining 11 939 records from the baseline review. The prompts were then run against the two updated reviews to test their validity.

Table 2 presents the prompts created during initial testing rounds. Prompt development and testing took place over 3 months (July–September 2024) and involved 1045 records. Changes to the initial prompt structure included the following (see online supplemental appendix D for a full description):

1. *Direct decision instruction*: adding “Return TRUE if…” to guide the LLM in selecting records that meet the criteria.
2. *Specific terms*: incorporating targeted terms, such as pharmacological interventions, to improve prompt precision.
3. *Hybrid question structure*: using formats such as “Does the study include [PICO element]?” improved accuracy by clarifying screening decisions. This format consistently reduced ambiguity in LLM responses and improved classification accuracy. Based on this finding, we recommend using yes/no questions when designing prompts for systematic review screening.

See table 2 for an account showing the progression of prompt development from the initial to the final version.

### Development, refinement and testing of prompts in the baseline review

Had the team developed prompts based on the 1045 records used in the evaluation and then applied them to the remaining studies in the baseline review (rather than screening those records manually), they would have reduced their workload by 65.72% (table 3). This would have meant they needed to screen an additional 3004 records in the LLM workflow, as opposed to the 11 939 that they did screen. This means that 3004 records were ‘reviewed’ manually after LLM filtering, compared with full manual screening of 11 939 in the baseline stage. The GPT-4o-based screening did not exclude any papers that were ultimately included in the review; however, it excluded 25 papers that the human team originally selected to assess based on their full-text reports. However, some records that human reviewers passed to full text were excluded by the LLM, reflected in the 0.58 sensitivity in Update 2 for full-text retrieval.

**Table 3:** Performance of the LLM in title/abstract screening with prompts

Title and abstract screening decisions						
	N to screen (human only)	N to screen (with LLM)	Workload reduction*	Sensitivity	Specificity	Precision†
Baseline	11 939	3004	65.72%	0.91	0.89	
Update 1	167	35	79.04%	1.00	0.87	0.04
Update 2	334	48	85.63%	0.58	0.92	0.20
Full-text decisions						
Baseline	11 939	3018	65.60%	1.00	0.88	
Update 1	167	35	79.04%	1.00	0.87	0.04
Update 2	334	48	85.63%	1.00	0.91	0.04

N = Number of records (or studies)

* Note that workload reduction is lower for the baseline because of the need to account for the fact that 1045 records were screened manually to provide data for prompt development.

† Precision not estimated for the baseline review, because of the way that included studies were ‘oversampled’.

LLM, large language models.

### Validation of prompts in the two update reviews

The same pattern applies when the two updates are considered. Again, the GPT-4o-based screening did not exclude any records that were ultimately included in the review updates, but in the second update, it did exclude five papers that human screeners chose to view at full text (but which were ultimately excluded). The simulated workload reductions were higher in the two updates than for the baseline review (at 79% and 86%, respectively), as they did not need to incur the ‘cost’ of manually screening records to develop the prompts.

### Consistency

The consistency of automated screening was assessed in two ways: (1) by running 50 randomly selected records through the GPT-4o API multiple times and (2) by testing 73 records previously included based on full-text appraisal. For the 50 random records, minor discrepancies were observed in exclusion codes (eg, ‘NOT depression’ being applied to some studies on some runs, but not on others), though inclusion/exclusion decisions remained stable across runs. Consistency for included records was higher, with only one erroneous exclusion, resulting in a 1/730 error rate. These findings indicate that while perfect accuracy in exclusion categories is unnecessary for this use case, the LLM demonstrates strong overall reliability in decision-making.

To quantify this reliability, we ran consistency checks on GPT-4o’s screening performance across multiple iterations. Online supplemental table 1 summarises the number of times each prompt code appeared across 10 reruns of 50 randomly selected records, as well as performance on 73 records that were included at full text. These results help assess whether LLM decisions remain stable when the same input is processed repeatedly; an important consideration for trust in automation.

## Discussion

### Summary of findings

This study developed and applied an iterative method for prompt refinement, testing and validation in a LSR. The process enabled the development of prompts that achieved high sensitivity and moderate specificity, suggesting they may be viable for use in future living review updates. However, while low precision values (4–20%) mean that some irrelevant records were still passed to human reviewers, these performance metrics suggest that the LLM could reduce the volume of records requiring manual review by nearly 80%. For researchers, this means fewer hours spent screening irrelevant studies, faster review updates and greater capacity to focus on complex tasks such as full-text screening or data extraction.^16 17^ Importantly, the high sensitivity obtained in this dataset (no missed eligible studies) indicates that this reduction in workload timesaving does not come at the cost of overlooking relevant evidence. While consistency on the precise exclusion criteria was less than 100%, the LLM showed strong overall consistency in determining study inclusion or exclusion. However, the adequacy of this accuracy depends on the review context, and further validation across additional LSRs is needed.

### Observations

We guided the LLM to adopt a step-by-step approach by testing exclusion criteria before evaluating studies against eligibility criteria, which are effectively the inverse of the exclusion criteria. This widely recommended method appeared to enhance the GPT-4o’s internal consistency, although it occasionally marked a record as both included and excluded.^18^ The LLM struggled most with very short abstracts, an issue we plan to examine further. While records with no abstracts were excluded from the assessment, extremely brief abstracts (eg, ‘This study will evaluate the effects of two antidepressant medications on sexual functioning’) may also need to be excluded from automated consideration to improve accuracy. We observed that abstracts under 20 words provided insufficient context for LLM screening. We recommend setting a minimum abstract length threshold or flagging these types of records for manual review.

Throughout the prompt development process, we found that explicitly instructing the LLM using Boolean formats (eg, “Return TRUE if…”), adding specific terms related to the PICO elements and framing questions clearly (eg, “Does the study include…”) all contributed to more accurate and stable outputs. Prompts that were too general or lacked clear guidance produced inconsistent classifications. These insights suggest that future prompt engineering should prioritise clarity, semantic grounding in eligibility criteria and robust handling of ambiguous abstracts.

Although exact time logs were not maintained, the team estimates indicate that approximately 30–40 hours of researcher time were required to refine and test the final prompt set iteratively. While we expect the roll-out of this approach in subsequent new reviews to take less time, the development process on this occasion required multiple refinement cycles and domain expertise, which highlights an important upfront cost in implementing these systems. Future evaluations should include data collection of time on task for this part of the work in order to factor it into workload estimation.

### Comparison to existing literature

Our findings align with prior studies highlighting the potential of LLMs in systematic review screening. Studies such as Guo *et al*,^8^ Krag *et al*^9^ and Cao *et al*^16^ reported high sensitivity and specificity for GPT-based models in similar tasks. While our study’s sensitivity for title/abstract decisions ranged from 58% to 100%, the key metric—whether eligible studies were missed—remained at 100%, ensuring perfect sensitivity. The observed specificity (87–92%) suggests that tailored prompt engineering enhances precision compared with standard prompts. Our iterative approach, which incorporated structured exclusion criteria, explicit instructions on borderline cases and hybrid question formats, likely improved precision by reducing ambiguous LLM outputs and improving decision consistency. However, further validation is needed to assess its generalisability across different review contexts. Improving precision remains a key area of active research. Possible strategies include refining prompts for borderline cases, incorporating structured metadata, incorporating the automated retrieval and assessment of full-text papers, ensembling, or combining LLMs with traditional machine learning (ML) filters. While our comparisons focused on other LLM-based approaches, future work should include direct benchmarking against more traditional screening methods, such as keyword-based heuristics or machine learning classifiers, using the same dataset. Future studies should benchmark LLM-assisted screening against more ‘traditional’ ML classifiers (eg, logistic regression, Support Vector Machines [SVMs] and Bidirectional Encoder Representations from Transformers [BERT]-based models) to evaluate their relative advantages and performance in systematic review workflows. This would help to contextualise the added value of prompt-based LLM workflows over simpler automation strategies, particularly in terms of sensitivity and workload reduction. (We note, however, that even with over 1000 records available to build the classifier, in this case, this did not result in a classifier that could compete with the LLM approach. An informal test using a logistic regression classifier resulted in workload reductions on the updates of only 40%: less than half that achieved by the LLM method.)

We did not collect data on how long it takes our research team to screen records, so it is difficult to quantify the workload saved in terms of person time (or costs). However, previous studies have shown that it takes between 2 and 5 min to screen studies for eligibility in systematic reviews. In our baseline review, this means screening all the records might have taken between 400 and 1000 hours, which might be reduced to between 140 and 350 hours using the LLM workflow.

### Strengths and limitations

A key strength of our study originates from the iterative development and testing of prompts that were tailored to the PICO framework of the systematic review. This approach allowed us to optimise the LLM’s performance for a specific task. Additionally, using a validation dataset rather than solely relying on the test set reduced the risk of overfitting, as it provided a more independent assessment of the model’s generalisability. Our findings suggest that this iterative prompt development strategy can yield prompts that may perform sufficiently well for integration into a ‘live’ review process.

However, this study is based on a single review using a single LLM with baseline data and two updates. Additional research may explore whether the prompt development strategy and screening performance translate to other LLMs such as Claude and Gemini, or open-source models such as Llama^19^ or DeepSeek.^20^ Differences in underlying architecture and training strategy may influence generalisability. Further testing across multiple reviews and using different LLMs will help determine whether the approach maintains high sensitivity and improves precision across different topics, datasets and LLMs. The claims about broader applicability should be interpreted cautiously. Replication across varied domains and PICO structures is necessary to evaluate generalisability. Moreover, we did not collect data on the length of time it took to develop the prompts, and future evaluations need to do this to estimate workload reduction accurately. Among other limitations, the LLM’s precision (4–20%) underscores the need for further refinement to reduce false positives, potentially through a workflow that incorporates full-text screening. Additionally, LLMs struggled to assess records with minimal or ambiguous abstract text, a limitation also observed in studies where incomplete information led to decreased screening accuracy and inconsistent decisions.^10 11^ Although hallucination is less likely in this kind of task, where the response is limited to responding true or false, we recommend incorporating safeguards such as manual audits, uncertainty scoring and human-in-the-loop designs to prevent erroneous exclusions. Addressing these challenges may require further advancements in LLM architecture or preprocessing strategies, such as integrating structured metadata or additional context from full-text records.

These limitations underscore the importance of cautious interpretation when integrating LLM-assisted screening into evidence syntheses. While high sensitivity helps ensure relevant studies are not missed, lower precision means that many irrelevant records may still need human review, limiting efficiency gains. Additionally, if models inconsistently handle ambiguous or brief abstracts, this could introduce variability into study inclusion decisions. Until these challenges are mitigated, human oversight remains essential to uphold the rigour and reproducibility of systematic reviews using LLM support.

### Implications for future research

The findings of this study have several practical implications. First, integrating LLMs into the screening workflow of LSRs can significantly reduce the time and effort required for human reviewers, enhancing the feasibility of maintaining up-to-date evidence syntheses. Second, the iterative prompt development process outlined here provides a scalable framework for optimising LLM performance in other review contexts. Third, developing software tools to systematically assess and enhance consistency at scale could further strengthen reliability.

Future research should assess the applicability of these methods across diverse review topics, including areas where structured data, such as animal studies and preclinical research, present unique challenges. Continued phases of this work will empirically assess workload reductions by logging time spent per record screened using the LLM-assisted approach compared with manual screening workflows. This will enable quantitative verification of the simulated reductions reported in this study. Additionally, integrating cross-format data—such as full-text articles and structured bibliographic metadata (eg, study design, intervention type)—could enhance screening accuracy by providing the LLM with richer contextual information. Further improvements in precision, whether through advanced prompt engineering, model fine-tuning or joint human–AI workflows, will be essential to maximising the reliability of LLM-assisted screening. To explore these strategies, we have begun developing prompts for two additional LSRs. For one of these (LSR6), test sets have been created from a subset of title and abstract screening records and continue to be refined and implemented in screening workflows.^21^ The LSR6 protocol includes plans to explore the use of machine learning (including LLMs) to support study selection and, potentially, data extraction, once the review enters living mode.^21^ One factor that will need to be considered is how well these approaches cope with ‘concept’ or ‘scope’ drift—when the focus or vocabulary used to describe eligible research changes. Moreover, newer LLMs may require substantially less prompt development and testing than was necessary in this use case.

It may be that, in the future, LLM-based study selection may involve the automatic downloading of full papers/reports and screening starting at this stage, rather than using titles and abstracts at all. However, current research in using LLMs for data extraction reveals that models struggle with complex study details and heterogeneous data types. Schmidt *et al*^22^ reported GPT-4 achieving ~80% accuracy in extracting data from clinical, animal and social science studies, though challenges persisted in capturing causal inference methods. Similarly, Claude V.2 demonstrated potential in extracting elements from randomised controlled trials, but accuracy varied across data types.^23^ LLMs also face inconsistencies in outputs, raising concerns about reliability and data contamination.^24^

Beyond improving efficiency, the use of LLMs in LSRs has important implications for research translation and evidence-based healthcare.^25^ By enabling more timely synthesis of emerging studies, this approach can support earlier identification of diagnostic innovations, emerging risk factors and targeted interventions. Faster updates to evidence summaries may also help prevent outdated findings from influencing clinical research priorities or public health strategies. Ultimately, more responsive and scalable review processes can strengthen the evidence base that informs prevention, diagnosis and treatment decisions.^26^

## Conclusion

This study demonstrates the potential of LLMs to support title and abstract screening in LSRs, achieving 100% sensitivity for ultimately included studies and an estimated workload reduction of ~80% for review updates. By applying iterative prompt engineering, we developed a high-performing workflow that preserved inclusion accuracy while significantly reducing manual screening effort. Integrating LLMs into systematic review workflows could meaningfully enhance the efficiency of evidence synthesis, though challenges remain—particularly around data extraction, where current models struggle with complex study details and heterogeneous formats. Further research should focus on improving model reliability, refining prompt strategies and validating tools in real-world review pipelines across diverse topics and LLMs to fully realise the benefits of LLM-assisted synthesis.

## Supplementary material

10.1136/bmjment-2025-301762 online supplemental file 1

## Data Availability

All project files, including datasets and supplementary materials, are available on the Open Science Framework (OSF) at https://doi.org/10.17605/OSF.IO/53VBH.
