# Supplementary material for: Development and evaluation of prompts for a large language model to screen titles and abstracts in a living systematic review
Source: BMJ Ment Health. 2025 Jul 22;28(1):e301762. doi: 10.1136/bmjment-2025-301762 (PMC12306261; doi:10.1136/bmjment-2025-301762)
Supplement: online supplemental file 1 [file bmjment-28-1-s001.docx]

# Appendix A: prompting schema

The following is an example of the prompt used to send to the GPT4o API. It contains a simple instruction to the LLM to extract data into the JSON-formatted response. It then appends the user-created prompts using the structure: label: data_type // definition. There is no hard-coded limit to the number of human-generated labels that can be added, and we have used it successfully with more than 50 such labels. After the ‘systemPrompt’ is defined, the title and abstract of the record are appended. Only one title and abstract (plus the ‘systemPrompt’) is submitted at a time.

systemPrompt = '''You extract data from the research information provided below into a JSON object of the shape provided.

If the data is not in the text, return "false" for that field. \nShape:

{non_human_study: boolean // this abstract does NOT report a study conducted with human participants., \n

Human_pharma_depression_study: boolean // this abstract reports the results of a randomized controlled trial; with human participants; evaluating a pharmacological treatment; examining dopamine pathways; the abstract does NOT state that participants have a diagnosis of schizophrenia or postpartum depression; the abstract states that the trial had a placebo group.}'''

// The title and abstract of the study is appended next

# Appendix B: EPPI Reviewer Prompt Structure

The EPPI Reviewer system enables users to build up sets of prompts for determining the eligibility of studies in a cumulative way. Users can create codes (for example, “NOT a human study”) that encapsulate a given inclusion or exclusion criterion. They then create the prompt using a specific syntax:

Label: data type // definition

(In this study, we have focused on the data type “Boolean”, as we simply wanted to know whether a given record does, or does not, meet a given eligibility criterion. Other data types include “string” - which extracts textual information, and “number” - for numeric data.)

In the example given above, the prompt may be made up of the label “non_human_study”, the data type “Boolean”, and the definition “this abstract does NOT report a study conducted with human participants”.

We depict below some example prompts (please see Appendix A for the technical definition):

- Exclude:
  - “NOT PICO element”
    - non_PICO_element: boolean // (description of PICO element exclusion)
- Include:
  - “PICO element”
    - PICO_element: boolean // (description of PICO element inclusion)

**Example prompts:**

1. Exclude:
   1. “NOT human study”
      1. Non_human_study: boolean // return TRUE if this abstract does NOT report a study conducted with human participants
2. Include:
   1. “INCLUDE title & abstract”
      1. Human_pharma_depression_study: boolean // return TRUE if this abstract reports the results of a randomized controlled trial; with human participants; evaluating a pharmacological treatment; examining dopamine pathways; the abstract does NOT state that participants have a diagnosis of schizophrenia or postpartum depression; the abstract states that the trial had a placebo group;

# Appendix C: Performance Metrics and Statistical Analysis

## Sensitivity

Sensitivity (also known as ‘recall’) is the proportion of relevant papers that the LLM identifies as relevant (include), and that the human screeners also identified to include. It is calculated by dividing the number of papers that the LLM included by the number of papers that the humans included.

## Specificity

Specificity measures the proportion of irrelevant papers that the LLM correctly identifies as irrelevant and excludes during the screening stage. This is calculated as the number of papers that the LLM identifies as excluded divided by the total number of papers excluded by humans in the manually screened dataset.

## Accuracy

To measure accuracy, the team assessed the overall correctness of the LLM’s screening compared to the human reviewer results. This was calculated as the total number of correct decisions made by the LLM (correctly included + excluded) divided by the total number of papers in the human reviewer screening dataset.

# Appendix D: Amendments to Prompt Structure for Optimization

## Direct decision instruction to the LLM: “Return TRUE if…”

This addition to the base prompt helped the LLM in processing the desired action by the group. It allows the LLM to select the marker “true” if the record is analyzed and meets the criteria for the prompt.

## Include specific terms which surpass the initial prompt description: “Including [specific term]”

When assessing the effectiveness of the prompts on the test dataset, the team incorporated terms found in the records that were applicable to the prompt and influenced the LLM’s decision. For example, including a specific pharmacological intervention in the prompt that may be missed by the LLM with the base prompt.

## Forming a hybrid prompt/question structure: “does the study include [PICO element]?”

The prompts were more effective in providing the LLM with the information needed to make a screening decision when formed as a question. By “asking” the LLM to identify whether the record meets the criterion, it leads to increased accuracy in screening decisions.

## Supplemental Table 1: GPT-4o consistency across repeated runs for inclusion/exclusion decisions and full-text includes

|  | Run (original is 0) | | | | | | | | | | |
| --- | --- | --- | --- | --- | --- | --- | --- | --- | --- | --- | --- |
| Consistency across 50 randomly selected records | 0 | 1 | 2 | 3 | 4 | 5 | 6 | 7 | 8 | 9 | 10 |
| NOT pharmacological and dopamine | 17 | 15 | 13 | 14 | 13 | 13 | 13 | 11 | 13 | 13 | 12 |
| NOT human study | 6 | 3 | 3 | 3 | 3 | 4 | 3 | 3 | 4 | 3 | 3 |
| NOT depression | 20 | 18 | 22 | 18 | 20 | 19 | 19 | 20 | 20 | 20 | 20 |
| NOT RCT | 18 | 15 | 16 | 17 | 15 | 17 | 17 | 18 | 16 | 16 | 16 |
| Systematic review | 10 | 10 | 10 | 10 | 10 | 10 | 10 | 10 | 10 | 10 | 9 |
| Diagnosis scz | 4 | 5 | 5 | 4 | 5 | 4 | 3 | 5 | 5 | 4 | 5 |
| NOT placebo-controlled | 27 | 24 | 25 | 25 | 24 | 25 | 24 | 26 | 25 | 26 | 24 |
| INCLUDE title & abstract | 8 | 9 | 10 | 8 | 9 | 9 | 8 | 8 | 10 | 7 | 8 |
| None of the codes above | 1 | 2 | 2 | 2 | 2 | 2 | 2 | 2 | 3 | 2 | 4 |
|  | | | | | | | | | | | |
| Consistency across 73 full text includes |  |  | | | | | | | | | |
| Exclude | 0 | 0 | 0 | 0 | 0 | 0 | 1 | 0 | 0 | 0 | 1 |
| Include | 66 | 63 | 62 | 64 | 65 | 63 | 67 | 61 | 63 | 64 | 63 |
| Not coded | 7 | 10 | 11 | 9 | 8 | 10 | 6 | 12 | 10 | 9 | 9 |
